# Supplementary material for: Bismuth antimicrobial drugs serve as broad-spectrum metallo-β-lactamase inhibitors
Source: Nat Commun. 2018 Jan 30;9:439. doi: 10.1038/s41467-018-02828-6 (PMC5789847; doi:10.1038/s41467-018-02828-6)
Supplement: Supplementary file 1 — Supplementary Information [file 41467_2018_2828_MOESM1_ESM.pdf]

Supplementary Information

Bismuth antimicrobial drugs serve as broad-spectrum metallo-β-lactamase inhibitors

Wang et al.

Supplementary Figures:

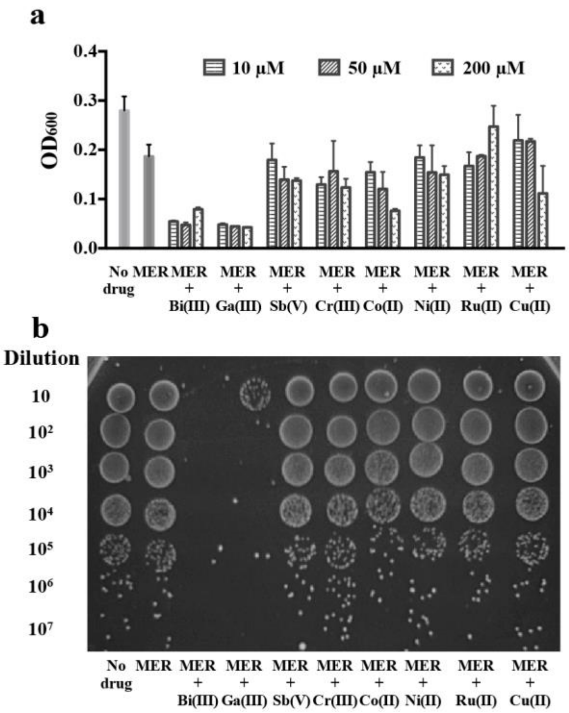

Supplementary Fig. 1 (a) Bar chart illustrating the growth inhibition with or without the exposure to MER or combination of MER and different metal compounds at increasing concentrations. (b) Representative phenotypic characterization showing the antimicrobial activity of MER (at 8 μg mL^{-1}) in the presence or absence of different metal compounds (at 10 μM) against an NDM-HK. No observable antimicrobial activity was detected when the metal compounds above were used alone.

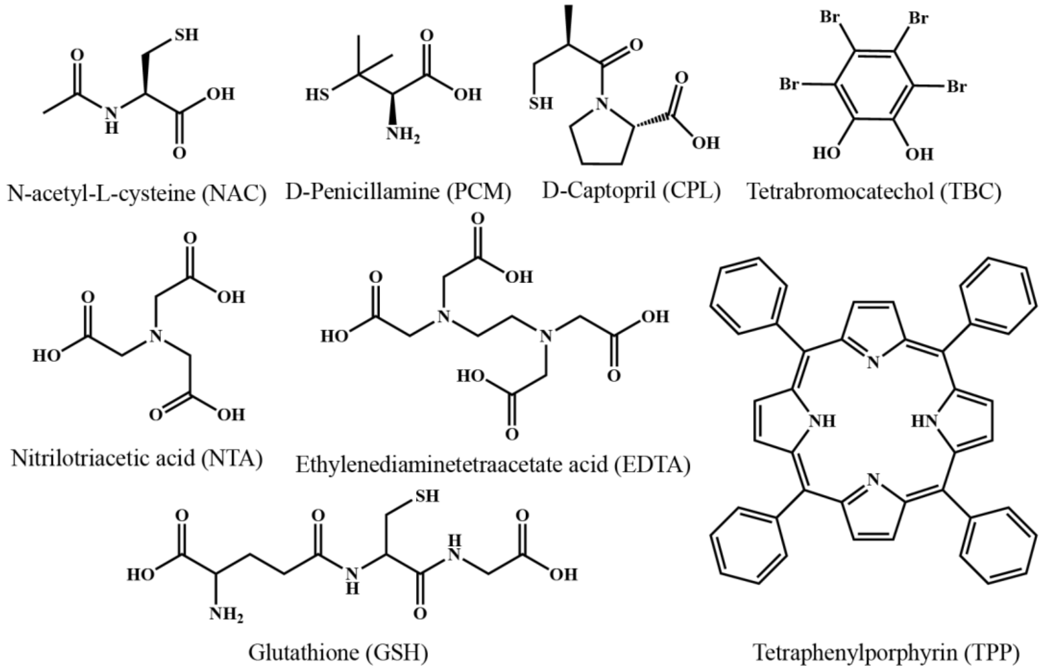

Supplementary Fig. 2 Chemical structures of the ligands used for preparation of Bi(III) compounds.

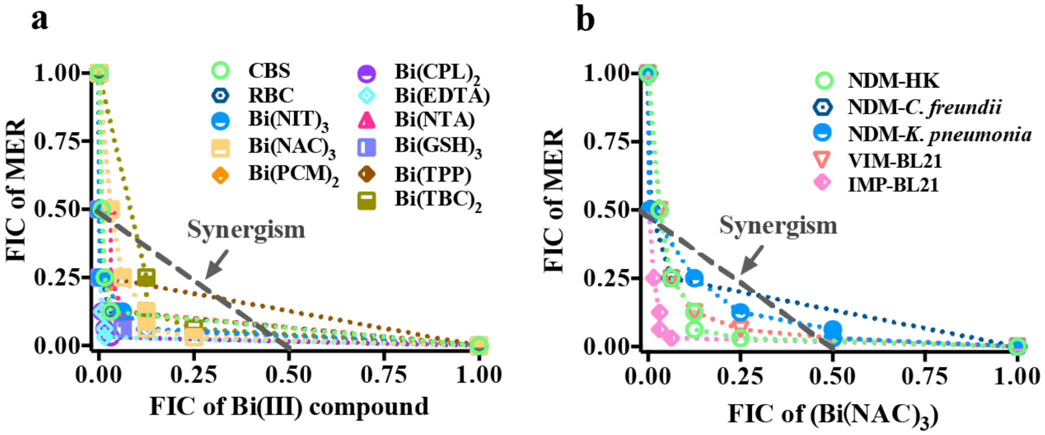

Supplementary Fig. 3 (a) Isobolograms for drug pairs of MER and different Bi(III) compounds against NDM-HK **(b)** Isobolograms for drug pairs of MER and $(Bi(NAC)_3)$ against different MBL positive bacterial strains.

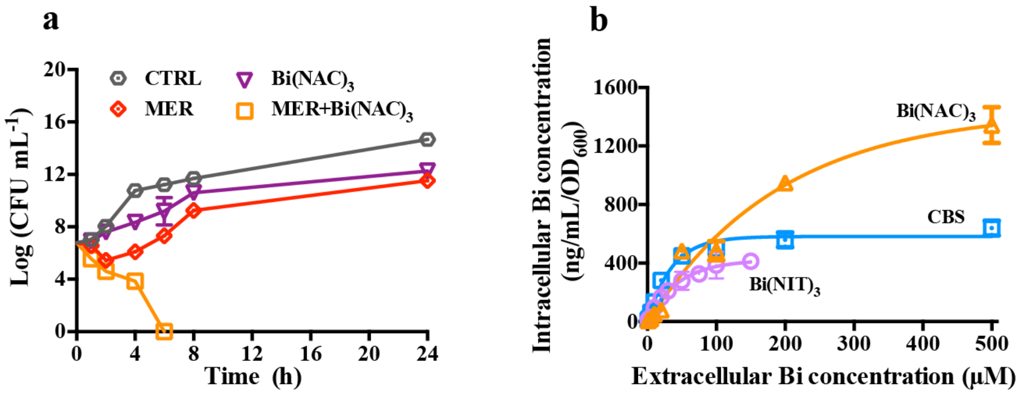

Supplementary Fig. 4 (a) Time kill curves for MER and ($Bi(NAC)_3$) monotherapy and combination therapy against NDM-HK during 24-hour incubation. The concentrations of MER and ($Bi(NAC)_3$) are 24 μg mL^{-1} and 32 μg mL^{-1} respectively. Mean value of three replicates are shown and the error bars indicate SD. **(b)** Concentration-dependent bismuth uptake in NDM-HK after supplement of CBS, ($Bi(NAC)_3$) and ($Bi(NIT)_3$) over a period of 24 hrs. Mean value of three replicates are shown and the error bars indicate SD.

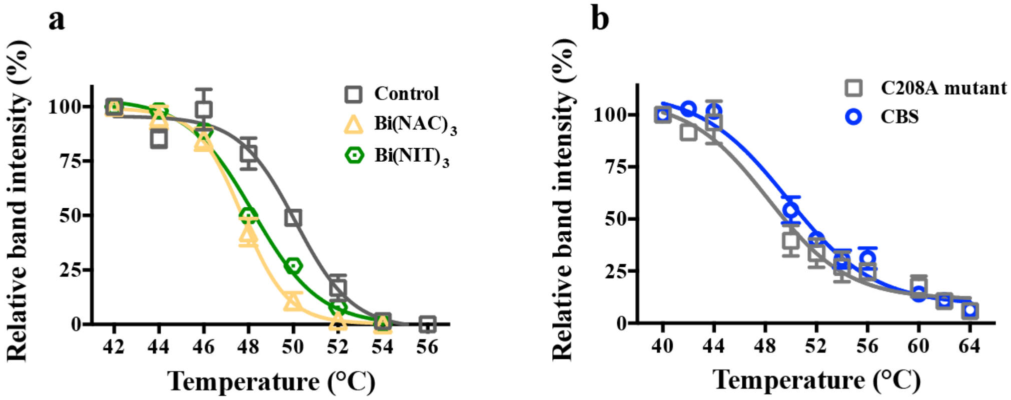

Supplementary Fig. 5 Cellular thermal shift assays showing **(a)** cellular target engagement of NDM-1 in NDM-HK when bacteria were treated with either ($Bi(NAC)_3$) or ($Bi(NIT)_3$). The corresponding melting temperatures were estimated to be 50.1 °C, 47.7 °C and 48.1 °C for control, $Bi(NAC)_3$-treated and $Bi(NIT)_3$-treated group respectively. Data are presented as means±SD from three independent experiments. **(b)** no obvious cellular target engagement in the mutant strain (C208A-Rosetta) treated with CBS. The corresponding melting temperatures were estimated to be 48.24 °C and 49.72 °C for the control and CBS-treated group respectively. The solid lines represent the best fits of the data to the Boltzmann sigmoid equation. Data are presented as means±SD from three independent experiments.

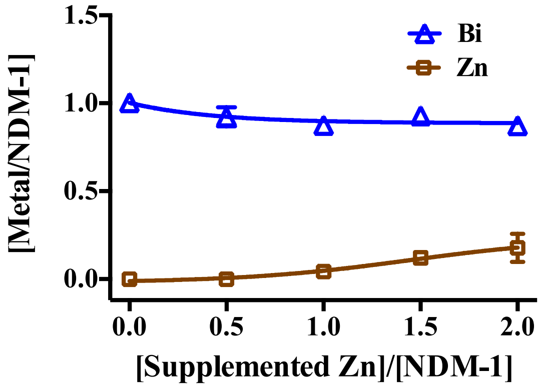

Supplementary Fig. 6 The metal contents upon supplementation of various amounts of Zn(II) into Bi-bound NDM-1. When two molar equivalents of Zn(II) relative to the protein was added, about 0.87 molar equivalents of Bi(III) still remained bound to NDM-1 protein, whereas *ca.* 0.17 molar equivalents of Zn(II) was also observed to bind to NDM-1, suggesting that only small portion of Bi(III) can be replaced by Zn(II) from NDM-1. Mean value of three replicates are shown and the error bars indicate SD.

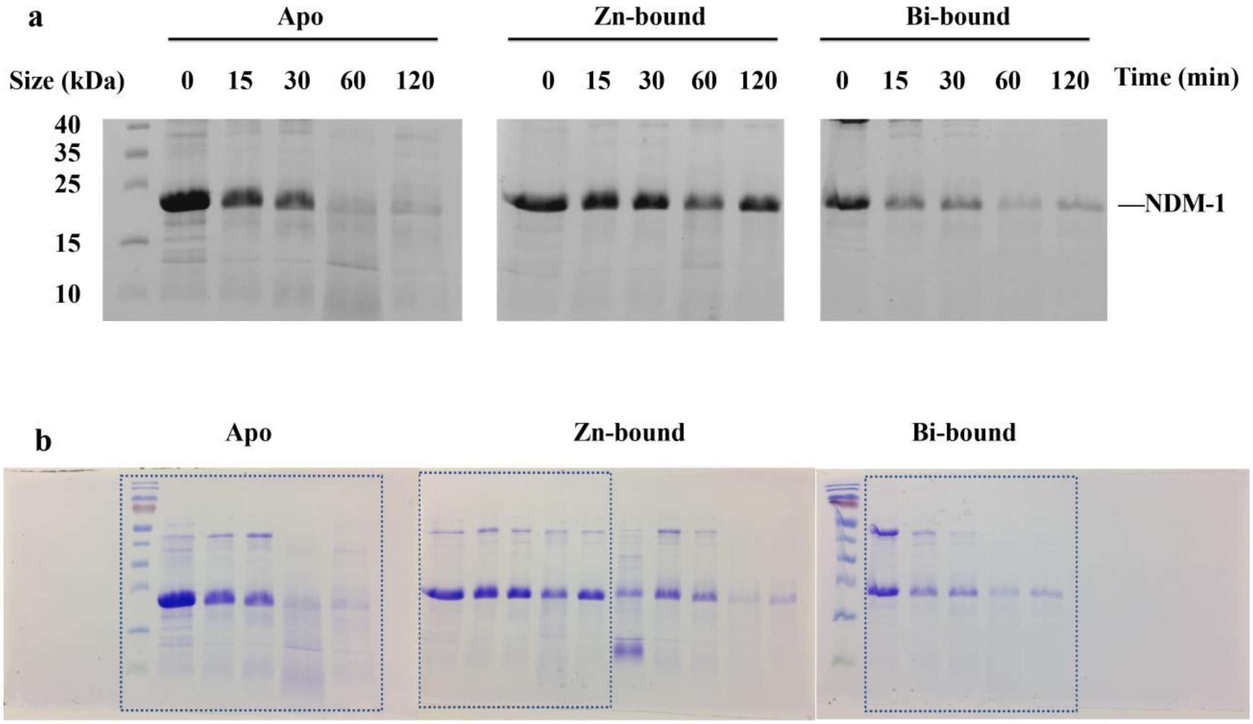

Supplementary Fig. 7 (a) Limited proteolysis of purified apo-, Zn-bound and Bi-bound NDM-1. Aliquots were taken at different time intervals and analyzed by SDS-PAGE. **(b)** Original image of SDS-PAGE gel.

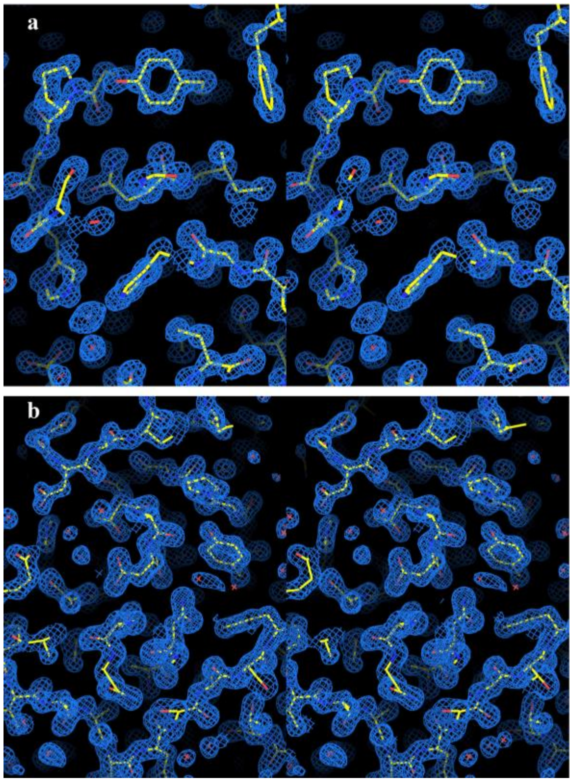

Supplementary Fig. 8 Stereo view of the 2Fo-Fc maps at 1.5 σ for (a) native Zn-bound NDM-1 and (b) Bi-bound NDM-1.

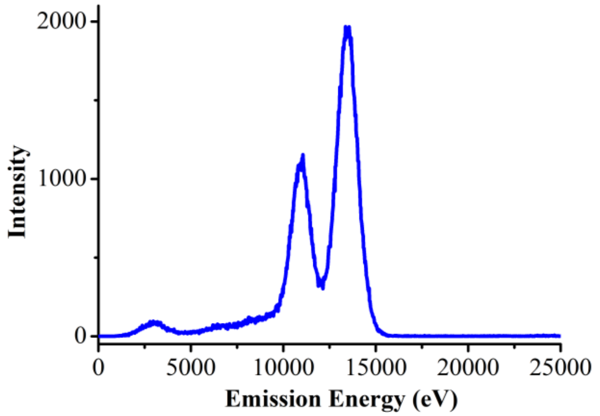

Supplementary Fig. 9 X-ray excitation scans at 0.92 Å. Bi exhibits emission at 10.8 keV, corresponding to L3 absorption edge. The emission at 13 keV corresponds to the excitation of Kr, which is the window material of the instrument.

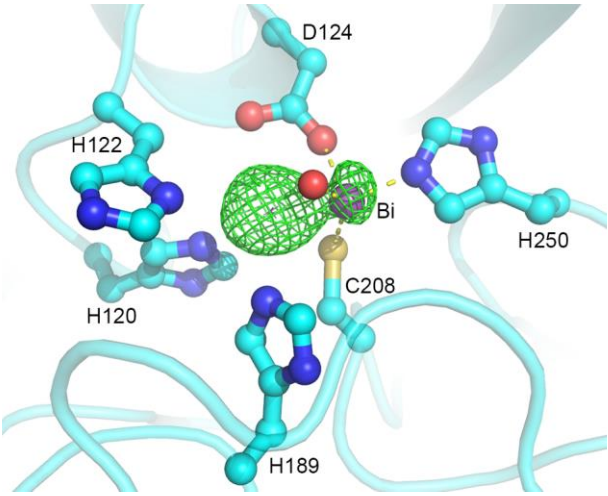

Supplementary Fig. 10 The active site of Bi(III) bound NDM-1 with Bi(III) occupancy of 0.1. The anomalous difference map was shown in green mesh and contoured at 5.0σ. Bi(III) coordinates His250, Cys208, Asp124 and a water molecule with a distorted tetrahedron geometry.

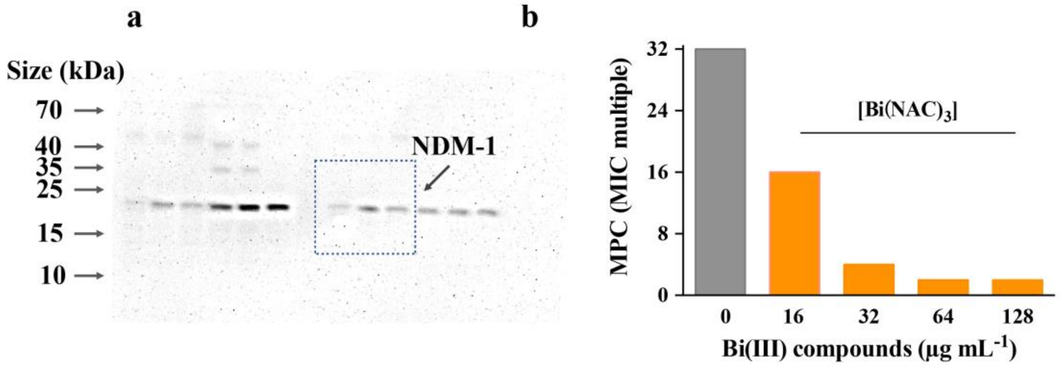

Supplementary Fig. 11 (a) Original image of western blot of NDM-1 expression level in **Fig. 4d (b)** Bar chart showing MPC values of MER in the presence of increasing concentration of ($Bi(NAC)_3$) against NDM-HK.

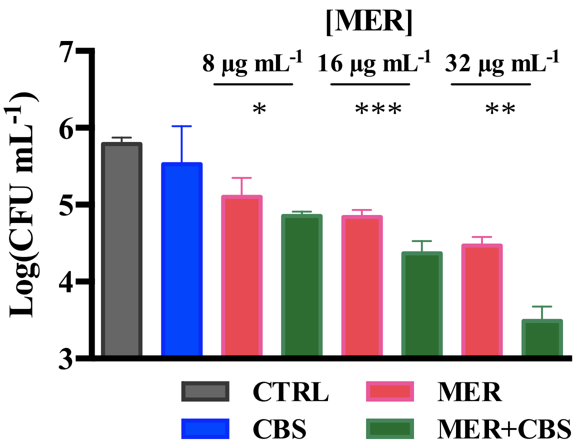

Supplementary Fig. 12 Bar chart showing the invaded-bacterial load in the *in vitro* infection model. The concentrations used are 8 μg mL^{-1}, 16 μg mL^{-1} and 32 μg mL^{-1} for MER and 32 μg mL^{-1} for CBS respectively. Mean value of three replicates are shown and the error bars indicate SD.

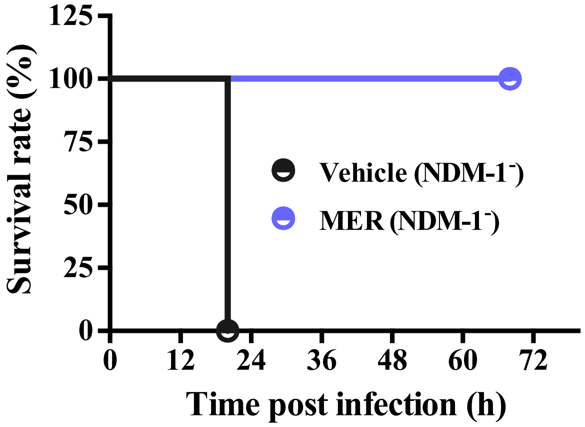

Supplementary Fig. 13 Survival curve showing efficacies in a murine peritonitis infection model. The NDM-1 negative bacterium NDM-HK PCV was used for infection. Five mice per group were used in both vehicle control and monotherapy of MER.

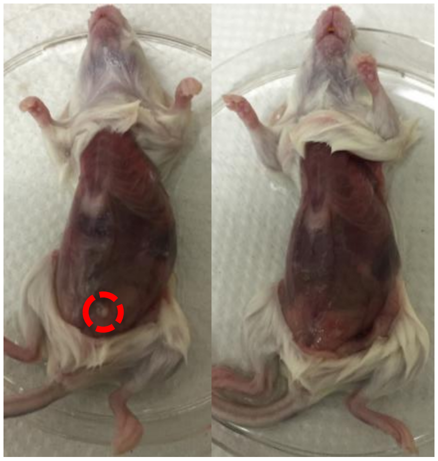

Supplementary Fig. 14 Comparison of dissected mice in the group of MER monotherapy (*Left*) and MER-CBS combination therapy (*Right*) showing that no bacteria were found in the group of combination therapy by visual inspection of the infection site.

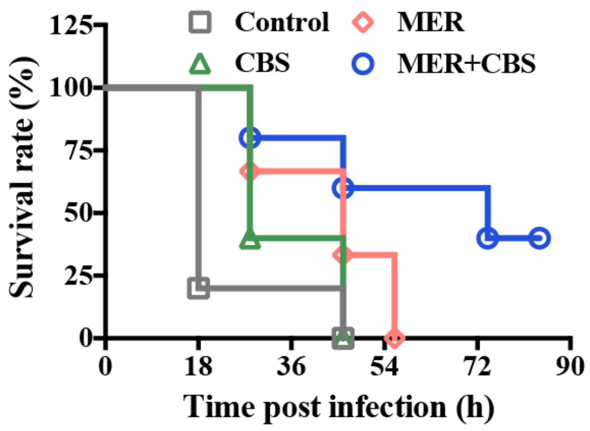

Supplementary Fig. 15 Survival curve showing efficacies in a murine peritonitis infection model without mucin. BALB/c mice (n=5 mice per group) were infected by a lethal dose of a clinical isolate of NDM-HK *via* intraperitoneal injection. Four groups of mice were treated with vehicle control, monotherapy of MER (50 mg kg^{-1}), CBS (50 mg kg^{-1}) or combination therapy of MER and CBS. P =0.0048, Mantel–Cox test.

Supplementary Tables

Supplementary Table 1: Bacterial information

Strain name	Relevant Genotype/Phenotype	Source
E. coli ATCC *25922*	Reference strain; MER^{S*}	In house
E. coli BL21(DE3)	Engineering strain; MER^{S}	In house
E. coli Rosetta(DE3)	Engineering strain; MER^{S}	In house
NDM-HK	Clinical isolate; NDM-1 positive; MER^{R*}	In house
NDM-HK PCV	NDM-HK variant cured of plasmid; MER^{S}	This study
NDM-BL21	*E. coli* BL21(DE3)(pET-28a-NDM-1); MER^{R}	This study
NDM-Rosetta	Rosetta(DE3)(pET-28a-NDM-1); MER^{R}	This study
NDM-Rosetta OX	NDM-Rosetta NDM-1 overexpression induced by IPTG	This study
C208A-Rosetta	NDM-Rosetta(DE3)(pET-28a-NDM-1-C208A)	This study
VIM-BL21	*E. coli* BL21(DE3)(pET-28a-VIM-2); MER^{R}	This study
IMP-BL21	*E. coli* BL21(DE3)(pET-28a-IMP-4); MER^{R}	This study
NDM-C. *freundii*	Clinical isolate of *citrobacter freundii*; NDM-1 positive; MER^{R}	In house
NDM-K. *pneumonia*	Clinical isolate of *klebsiella pneumonia;* NDM-1 positive; MER^{R}	In house

"MER^{S}" and "MER^{R}" stand for meropenem *sensitive* and *meropenem* resistant respectively

Supplementary Table 2: Susceptibility of MBL positive bacteria to the combination of MER and inhibitors

Strain	Compound	MIC/MBC ($\mu g\ mL^{-1}$)		
		Bi(III) alone	MER in combination with compound at 32 $\mu g\ mL^{-1}$	FICI
NDM-HK	MER alone	-	16/16	-
	CBS	>256/>256	2/4	0.250
	RBC	>256/>256	2/4	0.250
	$(Bi(NIT)_3)$	>256/>256	2/4	0.266
	$(Bi(NAC)_3)$	128/>256	0.5/0.5	0.188
	$(Bi(PCM)_2)$	>256/>256	2/2	0.313
	$(Bi(TBC)_2)$	>256/>256	1/2	0.250
	$(Bi(CPL)_2)$	>256/>256	0.5/0.5	0.125
	(Bi(EDTA))	>256/>256	0.5/0.5	0.094
	(Bi(NTA))	>256/>256	2/4	0.188
	$(Bi(GSH)_3)$	>256/>256	2/4	0.250
	(Bi(TPP))	>256/>256	4/8	0.375
	CIT	>256/>256	16/16	2.000
	CPL	>256/>256	16/16	2.000
	NAC	>256/>256	8/8	0.750
	EDTA	>256/>256	0.5/0.5	0.031
NDM-KP	MER alone	-	16/16	-
	CBS	>256/>256	4/8	0.375
	$(Bi(NAC)_3)$	256/>256	1/2	0.188
	$(Bi(NIT)_3)$	>256/>256	1/1	0.125
NDM-CF	MER alone	-	8/8	-
	CBS	>256/>256	0.5/1	0.125
	$(Bi(NAC)_3)$	256/>256	0.5/0.5	0.188
	$(Bi(NIT)_3)$	>256/>256	2/4	0.375
VIM-BL21	MER alone	-	32/32	-
	CBS	>256/>256	0.5/1	0.047
	$(Bi(NAC)_3)$	256/>256	0.5/1	0.063
	$(Bi(NIT)_3)$	>256/>256	1/2	0.063
	$(Bi(CPL)_2)$	>256/>256	0.5/1	0.078
	$(Bi(PCM)_2)$	>256/>256	1/2	0.093
IMP-BL21	MER alone	-	32/32	-
	CBS	>256/>256	4/8	0.187
	$(Bi(NAC)_3)$	256/>256	2/2	0.125
	$(Bi(NIT)_3)$	>256/>256	4/8	0.250

$(Bi(CPL)_2)$	>256/>256	4/8	0.287
$(Bi(PCM)_2)$	>256/>256	1/2	0.188

Supplementary Table 3: Susceptibility of wild type and mutants of NDM-1 strains against MER and CBS combination

Strain	MIC (μg mL^{-1}) of MER in combination with CBS (μg mL^{-1}) at			
	0	16	32	256
E. coli ATCC 25922	0.03	0.03	0.03	0.03
E. coli BL21(DE3)	0.03	0.03	0.03	0.03
E. coli Rosetta(DE3)	0.03	0.03	0.03	0.03
NDM-BL21	16	8	4	2
NDM-HK	16	4	2	2
NDM-HK PCV	0.03	0.03	0.03	0.03
NDM-Rosetta	32	8	4	4
NDM-Rosetta OX	256	128	128	32
C208A-Rosetta	0.12	0.12	0.12	0.12

Supplementary Table 4: Crystallographic data collection and refinement statistics

	Bi-NDM-1	Zn_2-NDM-1
**Data collection**		
Space group	$P2_1$	$P2_1$
Cell dimensions		
a, b, c (Å)	41.60, 60.18, 41.80	41.42, 59.81, 42.10
α, β, γ(°)	90, 98.95, 90	90, 97.78, 90
Resolution (Å)	50~1.55 (1.61~1.55)*	50~0.95 (0.98~0.95)
Unique reflections	27612 (2309)	127274 (12301)
Completeness (%)	95.5 (94.2)	99.5 (96.3)
Redundancy	7.0 (6.9)	6.9 (3.5)
Wilson B-factor	12.79	7.40
R_{merge}	0.116 (0.697)	0.094 (0.198)
$I / \sigma I$	18.1(3.1)	16.9 (4.8)
**Refinement**		
Resolution (Å)	50~1.55	50~0.95
R_{work}	0.1627 (0.2194)	0.1267 (0.1770)
R_{free}	0.1935 (0.2502)	0.1367 (0.1810)
No. atoms	1951	2167
Protein	1737	1806
Metal	1	2
Water	206	330
Protein residues	228	229
Ramachandran plot		
Favored (%)	99	99
Outliers (%)	0	0
Average *B*-factors	19.62	10.99
Protein	17.90	9.38
Water	33.77	19.62
Metal	14.94/18.42	7.25/6.42
Occupancy of metal ion	0.55/0.10	0.96/0.87
R.m.s. deviations		
Bond lengths (Å)	0.010	0.007
Bond angles (°)	1.37	1.21

*Values in parentheses are for the highest-resolution shell.
*Bismuth has two alternative conformations at the binding site.

Supplementary Table 5: Bond distances (Å) between metal ions and amino acid residues in the active site

	H_2O	H120	H122	D124-O1	D124-O2	H250	C208	H189
Bi1	2.64	2.65		3.02	2.70		2.72	2.85
Bi2	2.10				2.49	2.25	2.68	
Zn1	1.92	2.04	2.06					2.00
Zn2	2.55			1.98		2.06	2.32	

Supplementary Table 6: Mutation frequencies of resistance selection in NDM-HK

MIC Multiply	Mutation frequency when exposed to MER with CBS (μg mL^{-1}) at				
	0	32	64	128	256
0.5	$>1.31\times10^{-7}$	$>5.22\times10^{-8}$	1.23×10^{-8}	9.84×10^{-10}	1.31×10^{-9}
1	$>4.92\times10^{-8}$	4.10×10^{-8}	1.15×10^{-9}	—	
2	3.62×10^{-8}	3.28×10^{-9}	—		
4	4.43×10^{-8}	—			
8	3.12×10^{-8}				
16	—*				

* "—" represents that no mutant colony was observed

Supplementary Table 7: Primer sequences (5'-3')

Primer name	Primer sequence
NDM-1_NcoI_F	5'-GGGGGCCATGGGGTGAAATCCGTCCGAC-3'
NDM-1_EcoRI_R	5'-GGGGGGAATTCTTAACGCAGTTTATCAGCCAT-3'
NDM-1_C208A_F	5'-CGCGTTCGGCGGTGCCCTGATTAAAGATAGTAAAGC-3'
NDM-1_C208A_R	5'-GGCACCGCCGAACGCGATGTCGGTGCCATCAATGCCG-3'
VIM-2_NcoI_F	5'-GGGGGCCATGGGGAGCCCGCTGGCGTTTAGCGTG-3'
VIM-2_EcoRI_R	5'-GGGGGGAATTCCACCACGCTGCGGTTGGTATGCG-3'
IMP-4_NcoI_F	5'-GGGGGCCATGGGGAGCAAGTTATCTGTATTCTTT -3'
IMP-4_EcoRI_R	5'-GGGGGGAATTCTTAGTTGCTTAGTTTTGATGG-3'
